# Inhibition of 15-PDGH: a strategy to rejuvenate aged muscles?

**DOI:** 10.1186/s43556-020-00025-w

**Published:** 2021-05-20

**Authors:** Tianxia Lan, Xiawei Wei

**Affiliations:** Laboratory of Aging Research and Cancer Drug Targets, State Key Laboratory of Biotherapy and Cancer Center, National Clinical Research Center for Geriatrics, West China Hospital, Sichuan University, No. 17, Block 3, Southern Renmin Road, Chengdu, Sichuan 610041 P. R. China

In a study recently published in *Science*, A. R. Palla et al. identified a prostaglandin-degrading enzyme (15-PGDH) as a new therapeutic target for treating sarcopenia, and uncovered the mechanisms by which 15-PDGH impairs the aged muscles [1]. Sarcopenia is an age-related disease that causes the excessive loss of muscles. While the disease significantly affects patient’s balance, mobility and quality of life, no effective FDA-approved drugs are currently available. Notably, prostaglandin has been demonstrated as an important mediator of the regeneration and strength-augmentation of muscles [2], and the authors have previously observed that the levels of two members of prostaglandin family—prostaglandin E2 (PGE2) and prostaglandin D2 (PGD2) are decreased in aged skeletal muscles [3]. Thus, the major regulator of prostaglandin level, 15-hydroxyprostaglandin dehydrogenase (15-PGDH) was hypothesized to be implicated with the reduction of PGE2 and PGD2 in aged muscles.

To investigate the relevance between 15-PGDH and prostaglandin, A. R. Palla et al. performed 15-PGDH specific enzymatic activity assay, RNAseq as well as immunoblots in aged muscles, and comprehensively confirmed that the enzymatic activity and expression level of 15-PGDH were elevated. To further validate 15-PDGH’s role of diminishing prostaglandin level and thereby causing muscle atrophy, a gene therapy approach and a small molecular targeted drug were respectively used to inhibit 15-PGDH in muscles of young and aged mice. For the genetic approach, adeno-associated viral vectors were leveraged for the delivery of shRNA to 15-PGDH. Detected by liquid chromatography coupled to tandem mass spectrometry (LC-MS/MS), PGE2 and PGD2 levels were augmented following the knockdown of 5- PGDH in aged muscles. Moreover, the treatment of SW033291 (SW), a small molecule inhibitor of 15-PGDH also enhanced the PGE2 and PGD2 levels in aged muscles. Importantly, after the inhibition of 15-PGDH, the muscle mass of aged mice was more significantly increased comparing to that of young mice. And, according to the data provided by the authors, statistically significant increases of muscle force in both aged and young mice were also detected using a 305C muscle lever system. On the other hand, the authors overexpressed 15-PGDH gene in the muscles of young mice with an adeno-associated viral system, which is confirmed by qRT-PCR. As expected, the atrophy phenotypes were observed in young muscles. These findings indicate that elevation of 15-PGDH level is responsible for the lower prostaglandin level and contributes to sarcopenia. To determine the source of 15-PGDH in aged muscles, multiplex tissue imaging (CODEX) was used to identify the cells that express the enzyme in the aged *Tibialis anterior* (TA) and *Gastrocnemius* (GA) muscles. The significant co-localizations of 15-PGDH were found with myofibers and macrophages. Further, the myofibers and macrophages of aged muscles were respectively isolated for the detection of 15-PGDH (*Hpgd*) mRNA level by qRT-PCR. Substantial increases in 15-PGDH transcript levels of both cell types were detected, which confirmed that myofibers and macrophages are closely associated with the production of 15-PGDH.

As a matter of fact, it has been previously shown that PGE2 signaling is important for the muscle stem cell regeneration [2]. Hence, the authors managed to determine whether PGE2 is the major driver of the aged muscle regeneration following 15-PGDH inhibition. Therefore, SW and an AAV encoding the shRNA that targets the expression of PGD2 synthesizing enzyme were simultaneously injected into aged muscles, and successfully induced the regaining of mass, strength and endurance, indicating that increased PGE2 level can rescue the atrophy phenotypes in aged muscles independent of PGD2. Additionally, armed with Cre-lox system, the authors performed myofiber-specific ablation of the expression of the PGE2 receptor in aged mice, and found that inhibition of 15-PGDH could no longer benefit the muscles in aged mice. These two findings suggest that PGE2, but not PGD2, is responsible for the reversal of age-related muscle atrophy. However, an additional study analyzing the effects of PGE2 synthesizing enzyme gene knockout would make this part of research more integrated, as it is still possible that PGD2 makes minor contributions to sarcopenia. Further, to uncover the key signaling pathways involved in the PGE2-mediated muscle regeneration induced by 15-PGDH inhibition, the researchers analyzed the transcriptomes of SW-treated aged muscles using RNAseq. Among the pathways that are associated with age-related muscle atrophy, ubiquitin-related pathways and TGF signaling pathway were significantly reduced in aged muscles following the treatment of SW. Reportedly, PGE2 signaling could activate AKT pathways in non-muscle cells [4], and it might exert similar effect in muscle cells. Hence, the muscle cells were exposed to PGE2 in vitro to investigate downstream events of PGE2 elevation. An enrichment of phosphorylated AKT was shown by Western blots, and FOXO were inactivated. Moreover, the immunoblots also illustrated that the level of phospho-S6 ribosomal protein had also been increased, suggesting the augmentation of protein synthesis. Likewise, in vivo inhibition of 15-PGDH also leads to the increase of inactive phosphorylated FOXO. As the activation of FOXO is important for the improvement of muscle-specific atrophy-related E3 ubiquitin ligases’ expressions, the mRNA levels of these E3 ubiquitin ligases were consequently decreased, as shown by qRT-PCR. As for the TGF signaling pathway, the expressions of genes that are implicated in the age-related atrophy or dysfunction of muscle were also strikingly down-regulated after 15-PGDH inhibition [5]. Finally, the transcriptomic analysis of SW treated aged muscles also revealed strong elevations of a wide range of mitochondrial pathways, which indicate the improvements of content, function and morphology of the mitochondria in aged muscles. The increase of mitochondria content was confirmed by the increased mRNA level of a key regulator of mitochondrial biogenesis as well as the restored mitochondrial DNA level. In addition, the recovered mitochondrial function was demonstrated by the increased enzymatic activity of citrate synthase, succinate dehydrogenase staining and myofiber mitochondrial membrane potential. Furthermore, the improvement of mitochondrial morphology was observed under the transmission electron microscopy.

Taken together, the researchers showed that the inhibition of 15-PGDH in muscles of aged mice enhances the level of PGE2, a prostaglandin that contributes to the restoration of muscle mass and functions. Besides, the transcriptomic analysis of signaling pathways revealed strong declines in ubiquitin-related pathways and TGF signaling pathway, as well as a significant enrichment for mitochondrial pathways (Fig. 1) [1]. Their findings imply that 15-PGDH might be an ideal therapeutic target for treating sarcopenia. However, the network map of 15-PGDH signaling pathway still needs to be more comprehensively explored to ensure the efficacy and safety for the potential clinical applications.

**Fig. 1:**
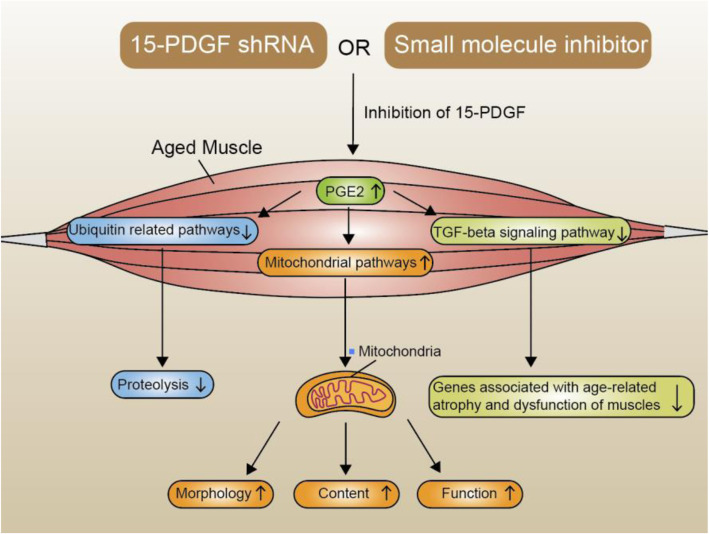
The signal pathways through which 15-PGDH inhibition induces the regeneration in aged muscles

## Data Availability

Not applicable.

## Abbreviations

15-PGDH: 15-hydroxyprostaglandin dehydrogenase
PGE2: Prostaglandin E2
PGD2: Prostaglandin D2
LC-MS/MS: Liquid chromatography coupled to tandem mass spectrometry
SW: SW033291
TA: *Tibialis anterior*
GA: *Gastrocnemius*
